# Skin-Lightening Product Use Among South Asian Americans: Cross-Sectional Survey Study

**DOI:** 10.2196/49068

**Published:** 2023-11-02

**Authors:** Manisha Banala, Anusha Mamidipaka, Temitayo Ogunleye

**Affiliations:** 1 Perelman School of Medicine University of Pennsylvania Philadelphia, PA United States; 2 Department of Dermatology University of Pennsylvania Philadelphia, PA United States

**Keywords:** colorism, skin-lightening products, skin bleaching, South Asian Americans, South Asian immigrants

## Abstract

**Background:**

Despite their potential for adverse health effects, skin-lightening products remain popular among South Asian Americans.

**Objective:**

This study investigates attitudes toward skin tone and the prevalence and adverse effects of skin-lightening product use among South Asian Americans.

**Methods:**

We conducted a cross-sectional study, recruiting and surveying 175 women or nonbinary individuals meeting the following inclusion criteria: (1) lived in the United States, (2) identified as South Asian, and (3) were raised by parents born in South Asian countries.

**Results:**

Of the 175 participants, 55 (31%) respondents used a skin-lightening product before. Parental pressure to use skin-lightening products and decreased time spent in the United States were significantly associated with skin-lightening product use (odds ratio [OR] 8.51, 95% CI 3.33-21.78, *P*<.001, and OR 0.70, 95% CI 0.52-0.96, *P*=.03, respectively). Although only 6 of the 55 (11%) users reported being aware of the potential side effects of skin-lightening products, 33 (60%) reported adverse effects, with acne, skin sensitivity, and dry skin being the most common. Users and nonusers equally endorsed statements associating lighter skin with increased attractiveness (*P*=.31), marriageability (*P*=.94), social status (*P*=.98), self-esteem (*P*=.73), and respect received from others (*P*=.74).

**Conclusions:**

The use of skin-lightening products among South Asian Americans is common and linked to social and psychological factors. Parental pressure and cultural beauty standards may play a significant role in perpetuating this practice. This study highlights the need for educational campaigns about the potential health risks associated with skin-lightening and increased efforts to challenge harmful beauty standards.

## Introduction

Fair skin is highly desired throughout South Asia, which encompasses Afghanistan, Bangladesh, Bhutan, India, Nepal, Pakistan, Sri Lanka, and the Maldives [1,2]. In India, skin-lightening products comprise 60% of the dermatological market [2]. Historical and cultural reasons exist for the popularity of these products throughout the region. During colonial times, British officers gave preferential treatment to light-skinned, high-caste South Asians, creating an association between light skin and economic prosperity [3]. To this day, India’s influential Bollywood film industry primarily features light-skinned actors who also star in advertisements for skin-lightening products [4].

South Asians continue to use skin-lightening products despite their well-documented adverse side effects [5]. One cross-sectional survey study conducted in a general medical outpatient clinic in Chhattisgarh, India, found that of 148 respondents, 30 (20%) had side effects from skin-lightening product use, most commonly acne (14%) and pruritus (5.6%) [6]. Another cross-sectional survey conducted in Kerala, India, found that of 306 skin-lightening product users, 185 (60%) had adverse effects, with 58 (19%) reporting burning sensations at sites of application and 29 (9.4%) experiencing increased dryness [7]. The most common active ingredients in skin-lightening products include hydroquinone, mercury, and corticosteroids [8]. These ingredients have been linked to systemic complications like adrenal insufficiency and membranous nephropathy and cutaneous complications like leukomelanoderma, exogenous ochronosis, squamous cell carcinoma, and dermatitis [4,9-11]. Despite regulations limiting the use of harmful ingredients in skin-lightening products, products often exceed legal limits or refuse to disclose them [4,12].

Immigrant communities originating from countries where skin bleaching is common often import the practice [13]. However, to our knowledge, there are no published studies investigating how South Asian Americans use skin-lightening products or how their parents influenced their perceptions of skin tone and skin-lightening products. This study aims to determine general attitudes toward skin tone and the prevalence of, motivations for, and adverse effects of skin-lightening product use among South Asians who lived in the United States.

## Methods

### Study Design

A cross-sectional study questionnaire was administered through Qualtrics, a web-based survey tool, with the following inclusion criteria: (1) lived in the United States, (2) identified as South Asian, and (3) were raised by parents born in South Asian countries [14]. The survey link was posted in a Facebook group titled “The Little Brown Diary,” a web-based community for South Asian women and gender minorities, and all members who met the inclusion criteria were asked to participate [15].

### Survey Overview

**Figure 1:**
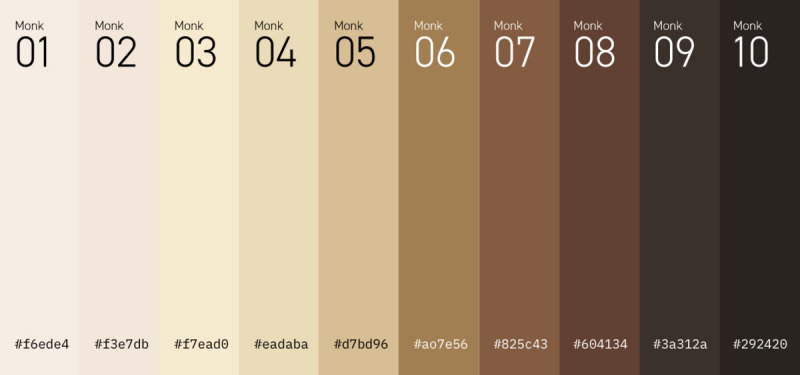
Monk’s 10-shade skin color scale.

Survey participants provided demographic data and indicated which skin tone in Figure 1 best matched their own. They were asked if they ever felt parental pressure to use skin-lightening products from their primary and secondary parent or if they ever felt unattractive due to their skin tone. “Primary parent” was defined in the survey as the parent who had the most influence on their upbringing. Participants also indicated if they lived in the United States for (1) less than 1 year, (2) 1-5 years, (3) 6-10 years, (4) 11-15 years, (5) 16-20 years, (6) 21-25 years, or (7) more than 25 years. A Likert scale was used to determine motivating factors associated with skin-lightening product use and asked participants to rate from strongly disagree (score 1) to strongly agree (score 6) the extent to which they felt skin tone impacted attractiveness, marriageability, social status, self-esteem, or respect received from others. The questionnaire was developed in accordance with information obtained from a literature review, and the content and validity of the questions were reviewed by a dermatologist at the University of Pennsylvania. Participants who reported past or current use of skin-lightening products provided information about the frequency of product application, location of application, and adverse effects experienced.

### Statistical Analysis

Statistical analysis of the survey responses was conducted with SPSS Statistics (version 29; IBM Corp). Descriptive statistics were used to summarize respondents’ reasons for using skin-lightening products. A 2-tailed independent samples *t* test was used to evaluate differences in motivating beliefs about skin-lightening product use between users and nonusers. A binary logistic regression model was used to assess factors associated with skin-lightening product use, using *P*=.05 as the cutoff for significance. Participants who did not answer all survey questions were excluded from the final model (n=10).

### Ethical Considerations

This study was reviewed by the University of Pennsylvania’s institutional review board and deemed exempt from requiring ethical approval.

## Results

### Study Population

Data from 175 completed surveys were analyzed. A total of 171 participants (98%) identified as women, and 4 (2.3%) identified as nonbinary. As shown in Table 1, a total of 96 (55%) respondents were between the ages of 20 and 30 years, and 53 (30%) were above the age of 30 years. A total of 110 (63%) respondents reported their highest level of education as a master’s degree and 62 (35%) reported a bachelor’s degree. The 175 respondents reported living across the United States. The most common states included New Jersey, California, and Texas, with 27 (15%), 25 (14%), and 14 (8%) respondents living there, respectively. The most common birth countries for respondents’ primary parents were India, Pakistan, and Bangladesh, with 151 (86%), 10 (5.7%), and 9 (5.1%) primary parents born there, respectively.

**Table 1:** Correlation between sociodemographic characteristics and use of skin-lightening products.

Variables		Users (n=55)	Nonusers (n=120)	Total (N=175)	OR^a^ (95% CI)	*P* value
**Age (years), n (%)**						
	18-20	8 (15)	18 (15)	26 (14.9)	Reference category	.16
	20-30	26 (47)	70 (58.3)	96 (54.9)	0.43 (0.13-1.48)	.18
	>30	21 (38)	32 (26.7)	53 (30.3)	1.03 (0.27-3.85)	.97
**Parents’ combined salary (US $), n (%)**						
	Not answered	2 (4)	7 (5.8)	9 (5.1)	Reference category	.70
	<100,000	27 (49)	46 (38.3)	73 (41.7)	1.87 (0.26-13.37)	.53
	100,000-250,000	21 (38)	53 (44.2)	74 (42.3)	2.66 (0.36-19.73)	.34
	>250,000	5 (9)	14 (11.7)	19 (10.9)	1.46 (0.13-16.16)	.76
**Education, n (%)**						
	Bachelor’s degree	21 (38)	41 (34.2)	62 (35.4)	Reference category	.10
	Master’s degree	32 (58)	78 (65)	110 (62.9)	0.58 (0.23-1.44)	.24
	Some college credit but no degree	2 (4)	1 (0.8)	3 (1.7)	9.69 (0.61-154.33)	.11
Duration lived in the United States, mean (SD)		6 (2)	6.3 (1.1)	6.1 (1.3)	0.70 (0.52-0.96)	.03
Felt unattractive, n (%)		33 (60)	60 (50)	93 (53.1)	0.69 (0.26-1.83)	.46
Pressure from primary parent, n (%)		34 (62)	18 (15)	52 (29.7)	8.51 (3.33-21.78)	<.001
Pressure from secondary parent, n (%)		9 (16)	2 (1.7)	11 (6.3)	4.02 (0.58-27.84)	.16
Skin tone, mean (SD)		5 (1)	4.8 (1.2)	4.9 (1.2)	0.84 (0.47-1.49)	.55
Primary parent’s skin tone, mean (SD)		4 (1)	4.5 (1.4)	4.3 (1.4)	0.85 (0.59-1.24)	.40
Secondary parent’s skin tone, mean (SD)		5 (1)	5.1 (1.4)	5.2 (1.4)	1.08 (0.74-1.57)	.68

^a^OR: odds ratio.

### Skin-Lightening Product Use and Adverse Effects

Of the 175 participants, 55 (31%) reported current or past use of a skin-lightening product. 43 (78%) of the 55 users' primary parents were born in India, 4 (7.2%) were born in Pakistan, and 4 (7.2%) were born in Bangladesh. Of the 55 users, 37 (67%) began using a skin-lightening product between the ages of 10 and 20 years, and 54 (98%) reported no longer using the product. The frequency of use varied, with 16 (29%) respondents using it daily and 9 (16%) only using it for special occasions. A total of 51 of 55 (93%) users reported applying the product to their faces, 12 (22%) on their arms, and 7 (13%) on their bodies. Although 33 (60%) participants experienced adverse effects, only 6 (11%) were aware of the potential adverse effects before they started using the product. The most reported adverse effects were dry skin, skin sensitivity, and acne, with 19 (35%), 15 (27%), and 9 (16%) participants experiencing them, respectively.

### Factors Motivating the Use of Skin-Lightening Products

As shown in Table 2, of the 55 respondents who reported current or past use of a skin-lightening product, 33 (60%) reported feeling unattractive at some point due to their skin tone. A total of 34 (62%) respondents reported feeling pressure from their primary parent to use the product, while 9 (16%) reported feeling such pressure from their secondary parent. Of the 55 skin-lightening product users, 51 (93%) identified their primary parent as their biological mother and their secondary parent as their biological father.

**Table 2:** Description of the use of skin-lightening products among respondents (N=175).

Variables		Frequency, n (%)
Still using product		1 (1.8)
Primary parent pressure		34 (61.8)
Secondary parent pressure		9 (16.4)
Ever felt unattractive due to skin tone		33 (60)
Awareness of side effects		6 (10.9)
Adverse effects from use		33 (60)
**Side effects**		
	Acne	9 (16.4)
	Skin sensitivity	15 (27.3)
	Dry skin	19 (34.5)
	Hyperpigmentation	3 (5.5)
	Hypopigmentation	3 (5.5)
**Areas applied**		
	Face	51 (92.7)
	Arms	12 (21.8)
	Body	7 (12.7)
	Hands	4 (7.3)
	Legs	5 (9.1)
	Feet	3 (5.5)
	Genitals	1 (1.8)
**Age when users started using product (years)**		
	<10	8 (14.5)
	10-20	37 (67.3)
	> 20	4 (7.3)
**Frequency of use**		
	Twice daily	4 (7.3)
	Once daily	16 (29.1)
	Every other day	3 (5.5)
	Once weekly	8 (14.5)
	Once a month	6 (10.9)
	Once yearly	1 (1.8)
	Only for occasions	9 (16.4)
	Once or twice ever	4 (7.3)
**Skin tone**		
	Light (A-C in Figure 1)	5 (9.1)
	Medium (D-G in Figure 1)	49 (89.1)
	Dark (H-J in Figure 1)	1 (1.8)

As seen in Table 1, significant associations were found between some sociodemographic characteristics and the use of skin-lightening products. Specifically, the duration the respondent lived in the United States and the pressure they felt from their primary parent to use a skin-lightening product were associated with skin-lightening use (*P*=.03 and *P*<.001, respectively). Respondents were about 9 times more likely to use a skin-lightening product if they felt pressured by their primary parent to do so (odds ratio [OR] 8.51, 95% CI 3.33-21.78). They were less likely to use a skin-lightening product the longer they lived in the United States (OR 0.70, 95% CI 0.52-0.96). Other sociodemographic factors such as age, parents’ combined salary, and education were not associated with skin-lightening product use. Notably, respondents’ skin tones were not associated with use. Additionally, as seen in Table 3, there was no significant difference between product users’ and nonusers’ understanding of how lighter skin influences attractiveness, marriageability, social status, self-esteem, or respect received from others.

**Table 3:** Reasons for skin-lightening.

Variables	Users (n=51), mean (SD)	Nonusers (n=114), mean (SD)	*F* test (*df*)	*P* value
Increase attractiveness	2.9 (1.4)	2.9 (1.2)	1.025 (163)	.31
Increase marriageability	3 (1.6)	2.8 (1.5)	0.006 (162)	.94
Increase social status	3.3 (1.4)	3 (1.4)	0 (163)	.98
Increase self-esteem	3.3 (1.4)	3.3 (1.3)	0.119 (162)	.73
Increase respect from others	3.4 (1.4)	3.3 (1.4)	0.109 (163)	.74

## Discussion

### Principal Results

This study aimed to estimate the prevalence of skin-lightening product use among self-identifying South Asians who lived in the United States. About 31% of the South Asian Americans surveyed reported current or past use of a skin-lightening product. This rate is greater than the previously reported skin-lightening product usage rate of 21% among the general United States population [17]. However, use of skin-lightening products appears more prevalent in North India (60%) than among South Asian Americans in the United States [5,6]. Pressure from a primary parent and decreased duration of residency in the United States were found to significantly predict the use of skin-lightening products. The findings of this study are unique in that they offer insight into how immigrant communities, specifically the South Asian community in the United States, import cultural practices.

A total of 33 of 55 (60%) users of skin-lightening products reported experiencing adverse effects. Surprisingly, only 6 (11%) users were aware of potential adverse effects, like acne, skin sensitivity, and dry skin, before applying the product. Earlier investigations conducted among South African and Southeast Asian university students demonstrated that 89% and 79% of participants, respectively, were aware of the potential side effects associated with using skin-lightening products. However, none of the participants in the South African study and only 30% of the Southeast Asian study could name the products’ active ingredients [18,19].

Interestingly, we did not find that education level or family income predicted skin-lightening product use. Despite the expectation that respondents with higher education levels would be more aware of the risks associated with bleaching products, the results showed no correlation between educational background and use. Like previous studies of populations in the United States and Saudi Arabia, this finding indicates that skin-lightening products are used by consumers of all educational backgrounds [5,20]. Additionally, skin tone did not predict skin-lightening product use. This finding is consistent with previous studies of adult women in Saudi Arabia and South Africa and suggests that skin tone also does not play a significant role in determining the use of skin-lightening products among South Asian Americans [18,20].

Like other studies, we found that skin-lightening product application often begins during users’ late teenage or early adult years, which may explain the lack of awareness of potential side effects [21]. A total of 37 of the 55 (67%) users began skin-lightening product application between the ages of 10 and 20 years, supporting the suggestion that early initiation of skin bleaching stems from identity development during adolescence [22]. Encouragingly, this study also found that 54 of the 55 (98%) respondents who once used a skin-lightening product no longer use it, which may suggest that cultural, antiracist movements promoting acceptance of dark skin positively influenced South Asian users of skin-lightening products. Other potential reasons for the high discontinuation rate include dissatisfaction with the product, side effects, unavailability, or a lack of affordability. The specific motivations for ending the use of skin-lightening products should be further investigated.

We did not find a significant difference between skin-lightening product users’ and nonusers’ understanding of how lighter skin impacts attractiveness, marriageability, social status, self-esteem, or respect received from others. Instead, we found a significant association between skin-lightening product use and parental pressure. Notably, respondents received pressure to use skin-lightening products more often from their mothers than their fathers. Since our respondents were mostly women, this proves previous studies’ findings that mothers are a key influence on their daughters’ body image [23]. Previous studies highlighted that familial socialization through parents' direct comments or indirect modeling relating to body image and attractiveness has a significant impact on children's self-esteem and their beliefs about attractiveness and body image [24,25]. Respondents should be conscious of how they speak about skin tone in front of their children to minimize the possibility of pressuring them to lighten their skin and perpetuating a cycle of poor self-esteem.

Finally, our research indicated a negative correlation between the duration of respondents’ residency in the United States and their use of skin-lightening products. While South Asian immigrants may have initially continued skin-lightening after moving to the United States, an extended period of living in the country appears to deter product use. Although no specific studies directly investigated this phenomenon, potential reasons for the decline in skin-lightening after moving to the United States include increased exposure to diverse ethnic and cultural backgrounds, decreased pressure to conform to South Asian beauty ideals, and limited availability of products.

### Limitations

There are several limitations to the methodology and findings of this study. First, the relatively small sample population was limited to self-identifying South Asian women and nonbinary Facebook group members who lived in the United States, which does not represent the entire South Asian American population. The relatively homogenous sample regarding gender, age, income, and education level could further limit conclusions. The survey relied on self-reported data, which may have introduced bias or misreporting. Moreover, the survey did not include questions about the specific skin-lightening products used by participants, which could have provided valuable insights into the prevalence and health risks associated with the use of these products. The survey also did not inquire about the number of products users applied or when or why they stopped using them. Given the high discontinuation rate, this would have added more depth to the research.

### Conclusions

In conclusion, skin-lightening remains a prevalent practice among South Asians who lived in the United States. This study sheds light on the attitudes and experiences of South Asian Americans and reveals that parental pressure encourages skin-lightening product use, but spending more time in the United States discourages it. Thus, parental pressure and South Asian cultural beauty standards may play a significant role in perpetuating this practice. This study highlights the need for educational campaigns about the potential health risks associated with skin-lightening agent use and increased efforts to challenge harmful beauty standards. Most skin-lightening product users were unaware of the products’ potential side effects, and dermatologists and regulatory bodies should work to illuminate these considerations and educate consumers. Further research is needed to better understand why most participants ceased use and to develop effective ways to discourage skin-lightening.
